# Intensifying cropping systems through doubled-up legumes in Eastern Zambia

**DOI:** 10.1038/s41598-021-87594-0

**Published:** 2021-04-14

**Authors:** Mulundu Mwila, Blessing Mhlanga, Christian Thierfelder

**Affiliations:** 1 Zambia Agriculture Research Institute, Msekera Research Station, Chipata, Zambia; 2 Institute of Life Sciences, Scuola Superiore Sant’Anna, Piazza Martiri della Libertà 33, 56127 Pisa, Italy; 3 International Maize and Wheat Improvement Centre (CIMMYT), Harare, Zimbabwe

**Keywords:** Plant sciences, Environmental sciences

## Abstract

Declining soil fertility and negative impacts of climate effects threaten the food security of millions in Africa. Conservation Agriculture (CA) is a promising strategy to address these challenges. However, lack of viable economic entry points and short-term benefits for smallholders limit its adoption. Legume intensification can possibly increase the output per unit area, thus making the system more attractive. Rotations of maize with intensified legume systems were tested for three consecutive years under ridge and furrow (RF) tillage and CA to investigate: (a) increases in productivity of legumes and the subsequent maize crop; (b) changes in land equivalent ratios (LERs) and; (c) improved total system productivity. Results showed an increase in legume yields when growing two legumes simultaneously, leading to greater LERs (ranging between 1.13 and 1.29). However, there was only a significant season and not a main treatment effect as CA did not outperform RF in both phases of the rotation. Full populations of companion legumes improved overall system productivity, yielding 76.8 GJ ha^−1^ in a more conducive season while sole cropping of pigeonpea yielded only 4.4 GJ ha^−1^. We conclude that the doubled-up legumes systems have great potential to improve household food security when integrated into current smallholder farming.

## Introduction

Food and nutrition security in southern Africa are a major concern for millions of smallholder farmers. This has become particularly important in recent years as climate variability and change are increasingly affecting smallholder farming systems^1,2^. In addition, declining soil fertility is critical for farmers^3^ due to limited use of mineral fertilizers and their ability to cope with crop demands of their main staple food crop, maize (*Zea mays* L.)^4,5^.

During the last two decades, efforts have been made to sustainably increase current farming systems using Conservation Agriculture (CA) with improved maize-legume systems as intervention strategies^6–11^. CA is a crop management system based on three main principles: minimum soil disturbance, crop residue retention and crop diversification through rotation and/or intercropping^12,13^. However, research has shown that the simultaneous application of these three main principles is often not enough to have an impact on soil quality and productivity increase. Complementary practices are needed to augment CA systems under the conditions of southern Africa^14^. Such complementary practices may include adequate fertilizer application, timely weeding including improved weed control with herbicides, use of stress-tolerant varieties, and integration of shrubs or tree-based components, among others.

CA systems are currently adopted on approximately 180 M ha around the world, with the bulk being adopted in South and North America, Canada and Australia, mostly on large commercial farms^15^. The extent of adoption is significantly smaller for smallholder farmers in Africa with approximately 1.5 M ha under CA to date^15^.

One of the key constraints hindering successful application of CA systems is the lack of adoption of the principle of crop diversification through rotations and intercropping in southern Africa^16,17^. Maize monocropping, especially in land constrained environments, is very common and has led to nutrient mining, soil carbon depletion, increased exposure to new invasive pests and diseases and soil erosion^18^. One way to make CA systems more attractive and financially viable is to diversify and increase the output per unit area, which is a key principle of sustainable intensification^19^. Companion legume systems, also known as “doubled-up legumes systems”, have been promoted in Malawi in the last two decades in an effort to increase productivity from the same area of land^20,21^. The systems combine two legumes in the field, which are less competitive in their growth habit. Usually, groundnut (*Arachis hypogaea* L.), a grain legume and pigeonpea (*Cajanus cajan* Millsp.), a woody perennial legume which contributes significant quantities of nitrogen (N) through the production of biomass (leaves and stems), while producing edible grain, are commonly used as companion crops in maize-based systems^22^.

Research by Turner and Taylor^23^ shows that growing maize in rotation with doubled-up legumes is more productive than continuous maize monocropping and maize-pigeon pea intercropping especially under low fertility and limited mineral fertilizer use. For southern Africa, the lack of available vegetative biomass in rotational systems is an impediment to sustained soil fertility improvement. Rotations with soybean (*Glycine max* L.) or groundnut mean that there is very little decomposable biomass left once the crop is harvested, but this can be provided by the pigeonpea in the case of a doubled-up legumes system. This added advantage needs to be quantified to understand how much each crop and their combination would add to the total system yield.

Previous research from southern Africa has shown that soil organic carbon (SOC) increase in CA systems is closely linked to the level of crop diversification^24^ and its absence can often lead to no SOC increase^25^. The inclusion of pigeonpea in a rotational system, especially if the fibrous parts of the residues are retained on the soil surface, is therefore seen as very beneficial in maintaining enough ground cover and adding organic carbon to the soil.

In pursuance of the integration of the concept of doubled-up legumes in CA systems, a research study was designed for Eastern Zambia. The objective of this study was to assess the performance of doubled-up legumes systems under CA as compared to conventional tillage-based ridge and furrow (RF) practices with significant interest in the rotational effects of the crop management system on maize productivity.

Our hypotheses were: (a) the rainfall season in target areas affects crop management systems more than soil treatment; (b) doubled-up legumes systems yield more per unit area in a rotational system than sole legumes; (c) doubled-up legumes systems under CA have a greater actual and residual benefit on legumes, and maize planted after the legumes; (d) doubled-up legumes systems lead to greater total system yield than sole cropping.

## Results

### Rainfall

Rainfall records from the trial sites showed different patterns in the three cropping seasons (Figure S2). However, the across site variability was almost the same for all the seasons with coefficient of variances (CV) ranging from 20.1 to 21.9% (Figure S2). Average rainfall in the cropping season 2015/2016 ranged between 402 and 655 mm, which coincided with one of the strongest El Niño seasons on record (Figure S2a). Cropping season 2016/2017 had higher rainfall of 717–1202 mm, coinciding with a medium La Niña season (Figure S2b). Finally, cropping season 2017/2018 was characterized by a rainfall range of 495–800 mm (Figure S2c). The rainfall variation across the seasons was high with a CV of 34.5%. Cropping season 2015/2016 and 2017/2018 were further characterized by long dry spells (up to 13 days) and early tailing-off of rainfall at the end of March of each season.

### Effect of crop management systems, doubled-up legumes systems, and seasons on legume productivity

**Figure 1:**
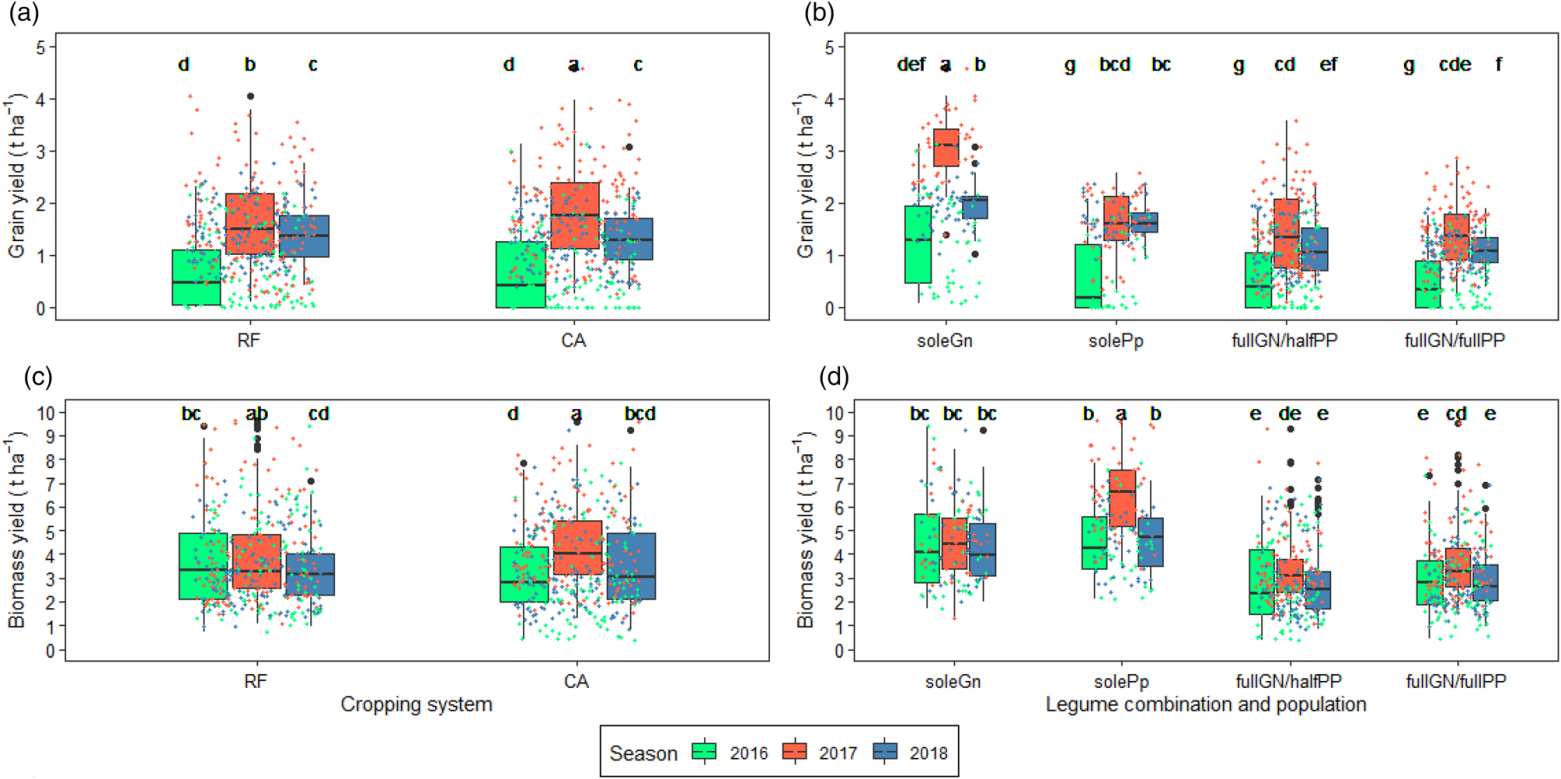
Interactive effects of different crop management systems and legume combinations and populations with seasons on (**a**), (**b**) legume grain yield and (**c**), (**d**) legume biomass yield over the seasons of implementation: RF = ridge and furrow; CA = conservation agriculture; soleGn = sole groundnuts; solePp = sole pigeonpea; fullGN/halfPP = legume intercropping with full population of groundnut and half population of pigeon pea; and fullGN/fullPP = legume intercropping with full population of groundnut and full population of pigeon pea. Boxplots with different letters above them are significantly different from each other. The error bars represent the standard error of mean (SEM). The jittered dots represent the individual observations from each plot over the seasons.

The interaction of crop management systems and years significantly affected grain yield of legumes (*p* < 0.0001, Table 1). The CA system in the 2016/2017 season had the highest grain yield averaging 2.0 t ha^−1^ (Fig. 1a). The least grain yield was observed in both the CA and the RF systems in the 2015/2016 season with an average of 0.73 t ha^−1^. In addition, there was a clear grouping of performance of the crop management systems based on the year with the crop management systems performing best in 2016/2017, followed by 2017/2018 and lastly 2015/2016. Additionally, legume grain yield was significantly affected by the interaction of the different legumes and their populations with seasons (*p* < 0.0001, Table 1). Sole groundnut in 2016/2017 had the highest grain yield of 3.1 t ha^−1^ (Fig. 1b). The least legume grain yield was observed in 2015/2016 for the fullGN/halfPP, solePp and fullGN/fullPP sub-treatments which averaged 0.5 t ha^−1^.

**Table 1:** Significance of fixed effects on legume and maize grain and biomass yield across the years.

Source	Degrees of freedom	Grain			Biomass		
		Sum of squares	Wald statistic	*p* value¶	Sum of squares	Wald statistic	*p* value¶
**Legumes**							
(Intercept)	1	113.89	324.15	2.20 e−16***	443.81	219.89	2.20 e−16***
Treatment (Treat)	1	0.807	2.3	0.12959^ns^	0.53	0.26	6.09 e−01^ns^
Subtreatment (Subtreat)	3	139.192	396.16	2.20 e−16***	670.93	332.43	2.20 e−16***
Season	2	172.551	491.11	2.20 e−16***	115.77	57.36	3.50 e−13***
Treat × subtreat	3	0.17	0.48	0.92236^ns^	26.04	12.9	4.86 e−03***
Treat × season	2	5.469	15.57	0.00042***	33.47	16.58	0.00025**
Subtreat × season	6	32.568	92.7	2.20 e−16***	66.51	32.96	1.07 e−05***
Treat × subtreat × season	6	0.216	0.62	0.99613^ns^	15.39	7.63	0.26684^ns^
Residual (MS)		0.351			2.02		
**Maize**							
(Intercept)	1	72.198	60.648	6.77 e−15***	154.853	137.16	2.20 e−16***
Treatment (Treat)	1	1.105	0.928	0.3354^ns^	0.768	0.68	0.40945^ns^
Subtreatment (Subtreat)	3	3.932	3.303	0.3473^ns^	7.948	7.04	0.07064. ^ns^
Season	1	71.86	60.364	7.88 e−15***	39.434	34.928	3.42 e−09***
Treat × subtreat	3	0.837	0.703	0.8725^ns^	0.794	0.703	0.8724^ns^
Treat × season	1	1.248	1.048	0.3059^ns^	0.041	0.037	0.84817^ns^
Subtreat × season	3	0.257	0.216	0.975^ns^	2.03	1.798	0.61531^ns^
Treat × subtreat × season	3	1.413	1.187	0.7561^ns^	5.301	4.695	0.19555^ns^
Residual (MS)		1.19			1.129		

^¶^^ns^, not significant.

*p < 0.05; **p < 0.01; ***p < 0.001.

For biomass yield, the interaction of crop management systems and seasons significantly affected legume biomass yield (*p* < 0.001, Table 1). The highest biomass yield was observed in the CA system in the 2016/2017 season and the same CA system yielded the least in the 2015/2016 season (Fig. 1c). The different legumes and their different populations significantly interacted with the seasons (*p* < 0.001, Table 1) and solePp had the highest yield of 6.5 t ha^−1^ observed in 2017 (Fig. 1d). The combinations of the legumes with full populations yielded the least in the 2015/2016 and 2017/2018 seasons with a mean yield of 2.8 t ha^−1^. Both legume grain and biomass yield differed across the years and across their different populations and combinations (*p* < 0.001 for both) (Table 1).

**Figure 2:**
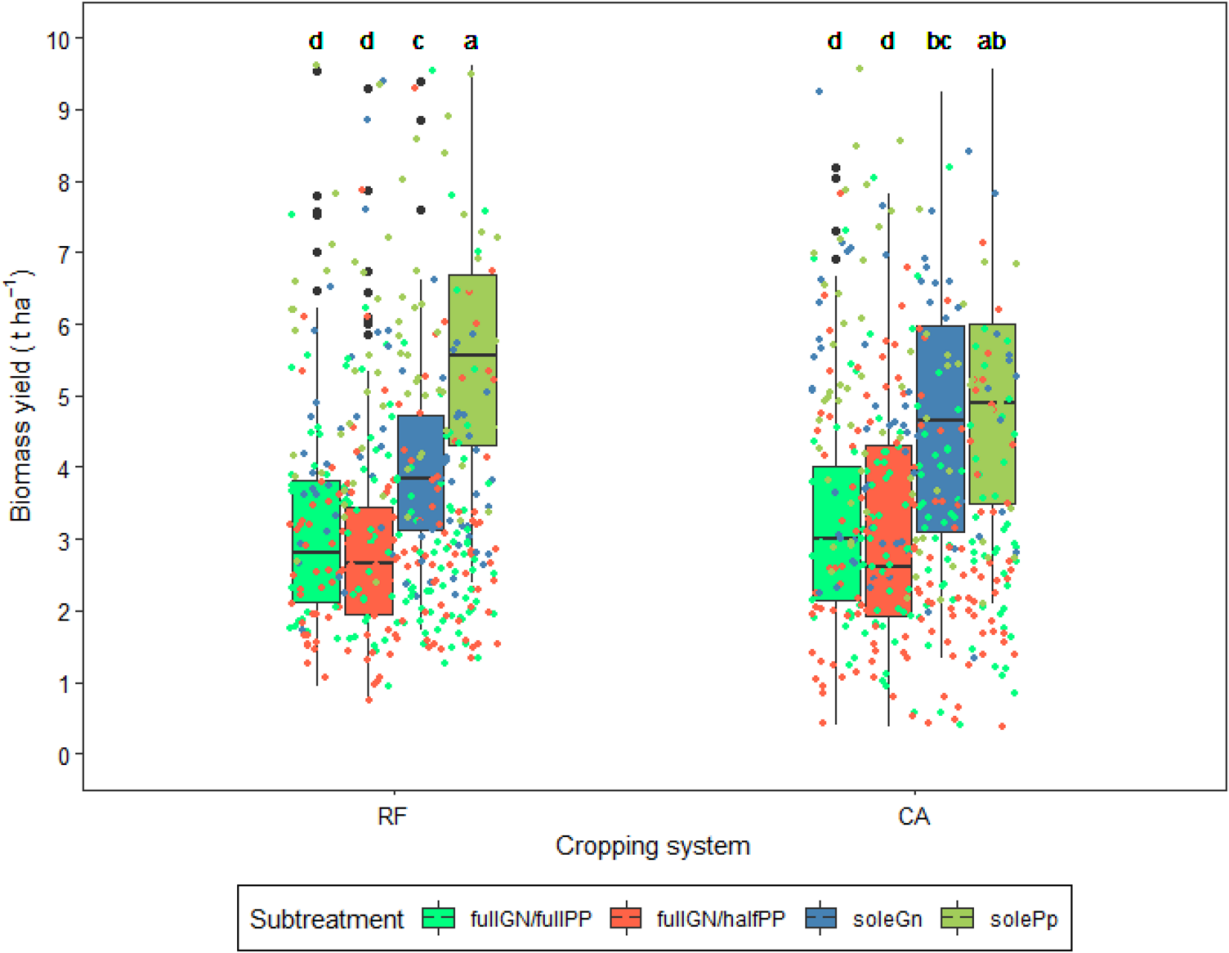
Interactive effect of crop management systems and legume combinations and their different populations on legume biomass yield over the seasons of implementation: RF = ridge and furrow; CA = conservation agriculture; soleGn = sole groundnuts; solePp = sole pigeonpea; fullGN/halfPP = legume intercropping with full population of groundnut and half population of pigeon pea; and fullGN/fullPP = legume intercropping with full population of groundnut and full population of pigeon pea. Boxplots with different letters above them are significantly different from each other. The error bars represent the standard error of mean (SEM). The jittered dots represent the individual observations from each plot over the seasons.

SolePp in RF systems resulted in highest yield of 5.6 t ha^−1^ but this was not significantly different from solePp under CA with a mean yield of 5.0 t ha^−1^ (Fig. 2). All the combinations of legumes and their populations yielded the least regardless of the crop management system in which they were combined and these averaged 3.0 t ha^−1^.

### Effects of crop management systems, doubled-up legumes systems, and seasons on maize productivity

**Figure 3:**
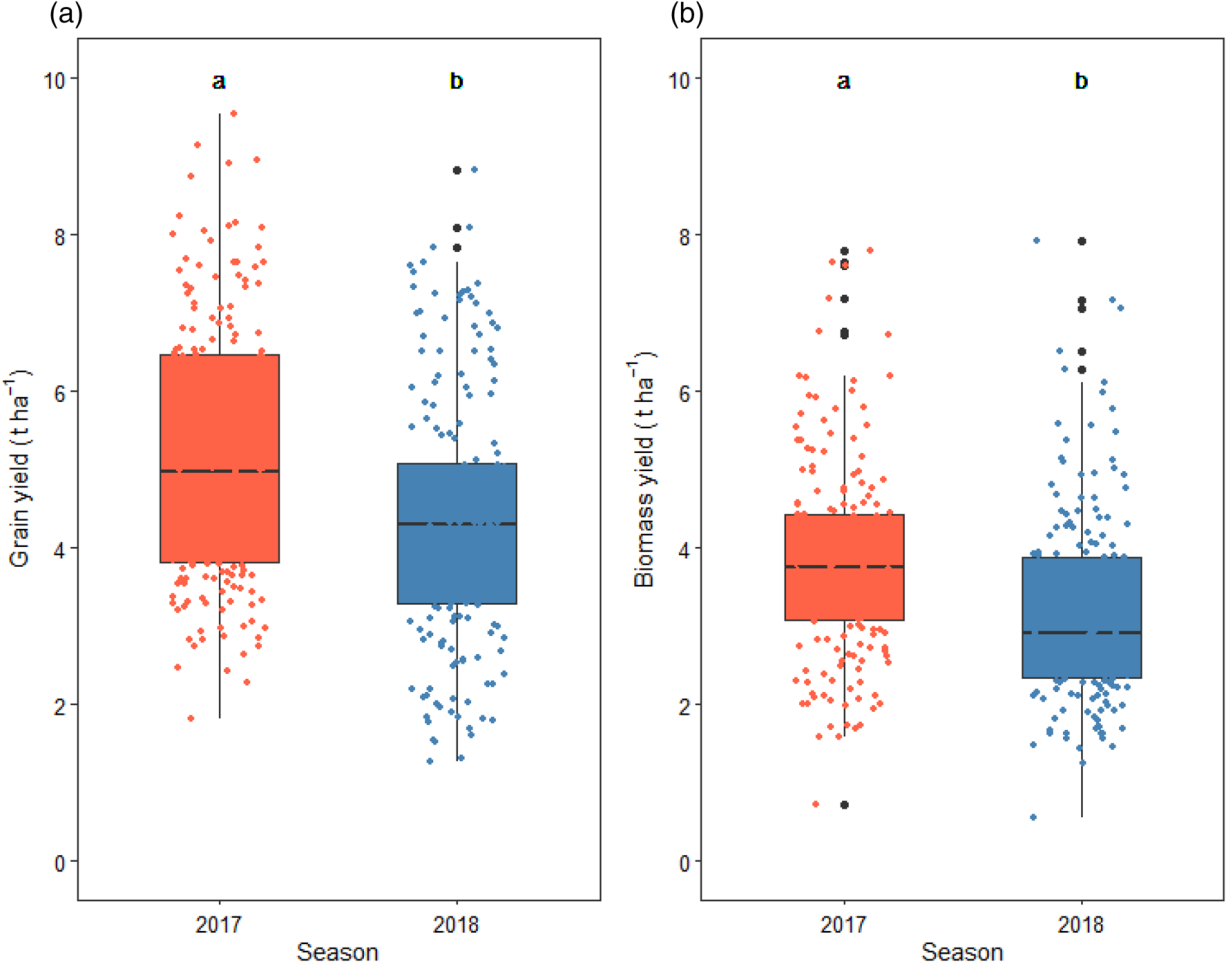
Effect of different seasons on (**a**) maize grain and (**b**) biomass yield averaged across all sites. Boxplots with different letters above them are significantly different from each other. The error bars represent the standard error of mean (SEM). The jittered dots represent the individual observations from each plot over the seasons.

Maize grain yields differed significantly only among the 2 years in which maize was planted (*p* < 0.0001, Table 1). Greater maize grain yield was observed in the 2016/2017 season and this had a mean of 5.2 t ha^−1^ while the 2017/2018 had a grain yield of 4.3 t ha^−1^ (Fig. 3a). Maize biomass yields also significantly differed among the years (*p* < 0.0001, Table 1). The 2016/2017 season had a greater yield of 3.8 t ha^−1^ while the 2017/2018 season had a yield of 3.1 t ha^−1^ (Fig. 3b).

Planting maize after the different legumes and their different population combinations was of marginal significance to maize biomass yield (*p* < 0.100, Table 1). Although the effect of these legumes was not significant, this marginality could reflect potential of significant effects with soleGn showing its high potential.

### Effects of different legume combinations and populations on total system grain, total system biomass and overall total system yield

In a two-phase rotation, both crops are grown within the same season although on half of the piece of land. Here, we assessed the contribution of all crops involved in a rotation as a measure of total system productivity in terms of energy expressed in GJ ha^−1^. However, we assessed total grain energy and biomass energy separately since these usually have different uses within a household and added them to get the overall system productivity.

**Figure 4:**
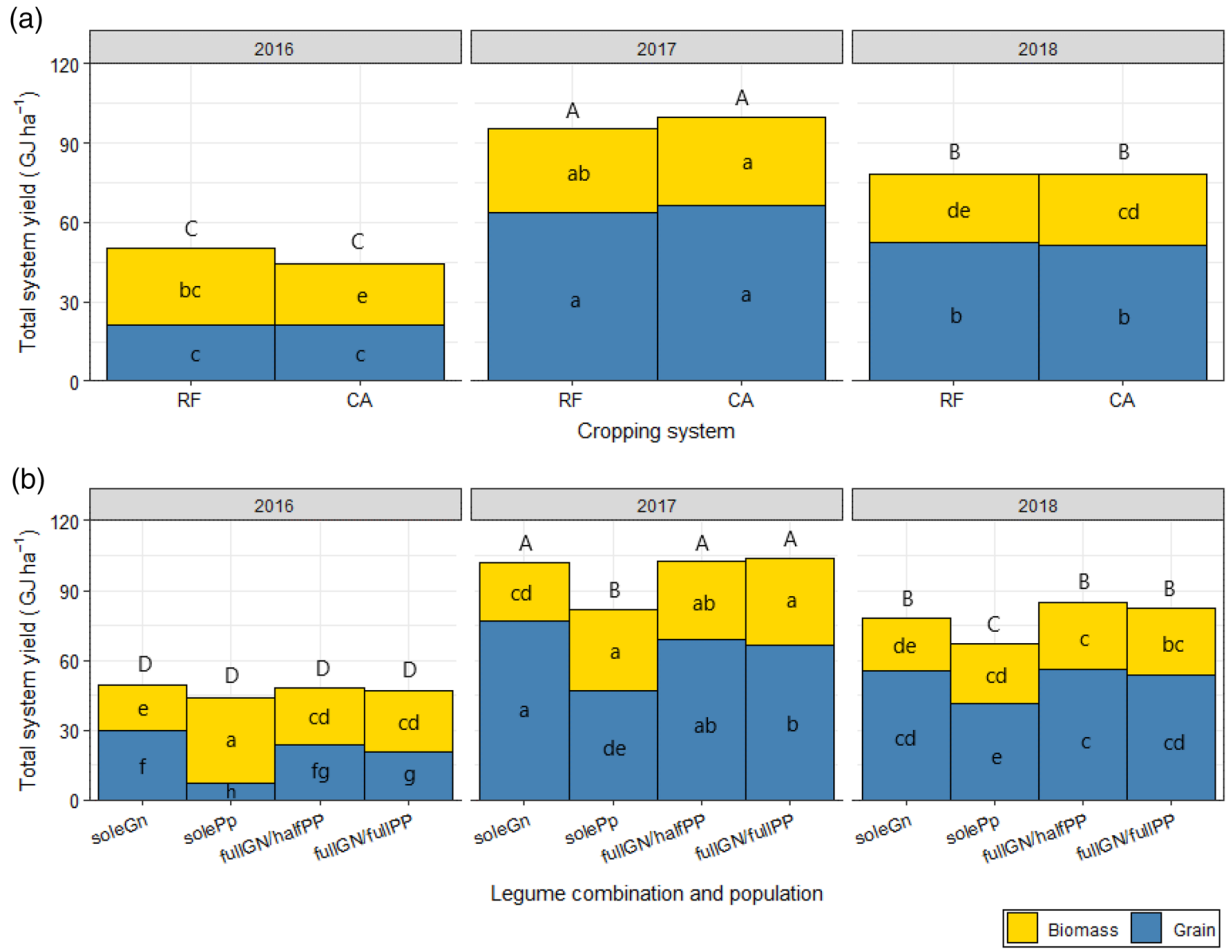
Interactive effects of (**a**) different crop management systems and (**b**) legume combinations and populations with the seasons on total system grain yield, total system biomass yield and overall total system yield (grain plus biomass) over the seasons of implementation: RF = ridge and furrow; CA = conservation agriculture; soleGn = sole groundnuts; solePp = sole pigeonpea; fullGN/halfPP = legume intercropping with full population of groundnut and half population of pigeon pea; and fullGN/fullPP = legume intercropping with full population of groundnut and full population of pigeon pea. The letters within each segment of the stacked columns denote significance for that segment for grain and biomass and the letters above the columns denote significance for overall total system yield. Stack segments and columns with different letters are significantly different from each other.

Overall total system yield was significantly affected by the interaction of crop management system and the seasons (*p* < 0.01, Table 2). The CA system in 2016/2017 season had the highest total yield averaging 99.8 GJ ha^−1^ and this was comparable to the RF system of the same season which averaged 95.5 GJ ha^−1^ (Fig. 4a). The least yield was obtained in the CA and RF systems both in the 2015/2016 season with an average of 46.2 GJ ha^−1^ (Fig. 4a). On the other hand, there was a clear grouping of performance based on the season as also observed for the individual crop performance. The legumes combinations affected total system yields differently (*p* < 0.05, Table 2). The highest total system yield was observed in 2017 for the fullGN/halfPP, soleGn and fullGN/fullPP subtreatments which averaged 102.8 GJ ha^−1^ (Fig. 4b). All different combinations of legumes and their populations in 2015/2016 resulted in the least total system yield ranging from 40.1 to 49.3 GJ ha^−1^.

**Table 2:** Significance of fixed effects on total system grain yield, total system biomass yield and overall total system yield across the years.

Source	Degrees of freedom	Sum of squares	Wald statistic	*p*-value¶
**Overall total system yield**				
(Intercept)	1	73,851	367.03	2.20e−16***
Treatment (Treat)	1	31	3.5	0.729655^ns^
Subtreatment (Subtreat)	3	17,395	225.63	1.35e−14***
Season	2	264,631	735.02	2.20e−16***
Treat × subtreat	3	508	0.75	0.57751^ns^
Treat × season	2	2633	26.28	0.005974**
Subtreat × season	6	3747	72.37	0.02384*
Treat × subtreat × season	6	708	3.31	0.839173^ns^
Residual (MS)		257		
**Total system grain yield**				
(Intercept)	1	31,484	198.04	2.20e−16***
Treat	1	56	0.35	0.554526^ns^
Subtreat	3	35,135	221	2.20e−16***
Season	2	208,213	1309.68	2.20e−16***
Treat × subtreat	3	144	0.91	0.824183^ns^
Treat × season	2	361	2.27	0.321296^ns^
Subtreat × season	6	3441	21.64	0.001405**
Treat × subtreat × season	6	363	2.29	0.891634^ns^
Residual (MS)		159		
**Total system biomass yield**				
(Intercept)	1	16,939.5	267.85	2.20e−16***
Treat	1	168.8	2.669	0.1023^ns^
Subtreat	3	7932.6	125.432	2.20e−16***
Season	2	5374.6	84.984	2.20e−16***
Treat × subtreat	3	232.8	3.682	0.57751^ns^
Treat × season	2	1999.8	31.621	0.005974***
Subtreat × season	6	3683.5	58.244	0.02384***
Treat × subtreat × season	6	395.5	6.254	0.839173^ns^
Residual (MS)		63.2		

^¶^^ns^, not significant.

*p < 0.05; **p < 0.01; ***p < 0.001.

Total system grain yield was significantly affected by the legume combinations, the seasons, and the interaction of legume combinations and seasons (*p* < 0.01, Table 2). Thus, we present in detail, the interactive effect of the legume combinations and seasons. Growing pigeonpea alone in the 2016/17 season resulted in the highest total system grain yield of 76.8 GJ ha^−1^ and this was comparable to growing groundnut in full population and pigeonpea in half population within the same year (Fig. 4b).

Planting sole pigeonpea resulted in the least total grain yield of 4.4 GJ ha^−1^. For total system biomass yield, the interaction of season and system (*p* < 0.001) and legume combinations (*p* < 0.001) significantly affected total system biomass (Table 2). The CA system in 2016/17 resulted in the highest total system biomass yield of 33.5 GJ ha^−1^ while the same system yielded the least in the 2016 season (23.3 GJ ha^−1^) (Fig. 4a). Planting pigeonpea and groundnut in their full populations in 2016/17, sole pigeon in 2016/2017 and sole pigeon pea in 2015/16 yielded highest total biomass with 37.3 GJ ha^−1^, 34.8 GJ ha^−1^ and 35.7 GJ ha^−1^, respectively (Fig. 4b). Sole groundnut in the 2015/2016 season resulted in the least total biomass yield.

### Land equivalent ratio

All doubled-up legumes systems had greater LERs than 1 indicating a yield advantage when growing two legumes simultaneously as opposed to growing one legume. When comparing monocropping to the legume configuration with both groundnut and pigeonpea at their full populations (fullGN/fullPP), LER significantly differed among the seasons (*p* < 0.01) (Table 3). The 2016/2017 and 2017/2018 seasons had the highest values of 1.3 compared to the 2015/2016 with an LER value of 1.1 (Table 4). There was no significant difference with the other population system (fullGN/halfPP) in terms of LER. When comparing monocropping system to fullGN/halfPP, LER values did not differ significantly among the systems, the seasons or their interaction (Table 3).

**Table 3:** Significance of fixed effects on land equivalent ratio (LER) of total system yield across the years.

Source	Degrees of freedom	Sum of squares	Wald statistic	*p*-value¶
**LER (FullGn/fullPp)**				
(Intercept)	1	104.074	2394.52	< 2.20e−16***
Treatment (Treat)	1	0.041	0.95	0.329709^ns^
Season	2	0.52	11.96	0.002524**
Treat × season	2	0.003	0.06	0.971593^ns^
Residual (MS)		0.043		
**LER (FullGn/halfPp)**				
(Intercept)	1	111.718	1542.15	< 2.00e−16***
Treatment (Treat)	1	0.02	0.27	0.6013^ns^
Season	2	0.312	4.31	0.1159^ns^
Treat × season	2	0.21	2.9	0.235^ns^
Residual (MS)		0.072		

^¶^^ns^, not significant.

*p < 0.05; **p < 0.01; ***p < 0.001.

**Table 4:** Land equivalent ratios for the different legume configurations for total system yield averaged across locations in different seasons.

Season	Land equivalent ratio	
	FullGN/fullPP	FullGN/halfPP
2015/16	1.13 b	1.29 a
2016/17	1.28 a	1.22 a
2017/18	1.25 a	1.16 a

Means followed by different letters are significantly different from each other.

## Discussion

Declining soil fertility and increased threats of climate variability and change will require that crop management systems are sustainably intensified or the number of hungry people around the world will increase^1,19,26^. We present one strategy of sustainable intensification of current maize-based cropping systems through incorporation of doubled-up legumes systems. The results of the trial are consistent with those of Snapp et al. (2002) in showing that combining two legumes in maize-legume rotations enhance crop yields on limited landholding^17^. Nutritional benefits from the inclusion of more legumes in the diet can be anticipated^27^. It can further support cash-constrained farmers who cannot buy large quantities of mineral fertilizers to maintain soil fertility through biological N fixation^28,29^.

The study focussed on answering four hypotheses which will be discussed here.

The relatively high CV in the rainfall observed over the study seasons shows how variable the rainfall patterns have become because of climate change and the associated risks that farmers face^30^. The long dry spells and early tailing-off of the rainfall seasons significantly affected crop production in the trial areas as it coincided with critical growth stages (pod formation on legumes and/or cob formation and grain filling on maize). From the statistical analysis (Table 1), the seasonal rainfall effect was stronger than the crop management systems effect. Our first hypothesis can therefore be accepted. The results call for crop management systems that are more adapted and more resilient to an increasingly variable climate^31^. Overcoming the downside effects of in-season dry-spells has previously been researched upon^32^ and CA holds promise to be climate-resilient^33^. Previous research from Eastern Zambia also showed that CA systems can maintain or increase their productivity despite these climate conditions^8,34^. Combinations of improved doubled-up legumes systems and CA therefore seem logical as this would reduce the impact of climate on the crop management systems.

All doubled-up legumes systems led to higher LERs which indicates advantages of growing several crops over monocropping grain legumes. In the 2015/2016 season, lower grain yields were recorded in fullGN/fullPP when compared to the 2016/2017 and 2017/2018 seasons as reflected in the lower LERs (Table 4). The lower yield of pigeonpea in the first season affected the crop management system in that season. The reasons were lack of control of pests and crop competition for soil moisture in the very dry El Niño year. Pigeonpea is frequently affected by insect pests and requires control as often the pests affect the reproductive parts (flowers, pods) as previously found out by^35,36^. As per the LERs, there was a clear advantage of growing crops in combination in a doubled-up legumes system thus supporting our second hypothesis. Interestingly, the other population (fullGN/halfPP) had the opposite trend of yielding more in the driest year (although not significant), whereas in other years it was not as productive. It appears that moisture stress and crop competition between the species was less pronounced in this population in the very dry years as compared with (fullGN/fullPP), where both factors could have been mitigated by higher rainfall in the other two seasons.

The lack of significant main treatment effect in the 3 years of study was surprising and unexpected. Possible reasons for the lack of a consistent response to the main crop management systems considering a variable climate may include the following: (a) the same plant population of groundnut was used in both CA and non-CA treatments to reduce the bias due to plant population. Traditionally, farmers in Eastern Zambia just plant a single row of groundnut on top of the ridge while double rows is a new cropping practice researched and promoted by the International Centre for Research in the Semi-Arid Tropics (ICRISAT) and its partners in the region^37^. The comparison with the traditional farmer spacing would have probably yielded significant effects of the crop management system as has been previously shown in Malawi^38^; (b) a relatively short-term conversion period (3 years) from RF to CA as researched in these trials did not lead to immediate yield benefits. This lag time until benefits accrue has been highlighted as a major disadvantage of the promotion of CA systems^10^. It normally requires 2–5 cropping seasons in a maize-based system until the yield benefits become apparent^39,40^. Our third hypothesis, that doubled-up legumes systems under CA increase the maize and legume yields was therefore partially rejected. However, the interaction of the crop management system and the season showed superior performance of CA in some seasons under a variable climate.

Labour requirements to plant crops on the flat and on ridges were not measured. We therefore suggest that even in the absence of a conclusive yield advantage over RF, CA would confer reduced labour for planting and weeding upon practitioners of the crop combinations studied. Producing a crop with less labour on planting, as no manual ridging is required under CA, would mean an immediate economic benefit for smallholders while waiting for the longer-term yield increase. However, as these benefits were not quantified in these trials we can only refer to other literature on economic benefits of manual CA systems in the region^8,41,42^.

The reduced legume grain yield for the fullGN/halfPP, solePp and fullGN/fullPP subtreatments in the 2015/2016 season, considering the legume combinations and seasons interactions may have been due to moisture stress experienced as a result of the low rainfall. This was the year of an unprecedented El Niño, which significantly affected the whole region and its farmers^43^. Competition for moisture in more intensified crop management systems with several crops in a doubled-up legumes system is expected and may have led to reduced productivity of the constituents^44,45^.

The reduced biomass yields in the doubled-up legumes systems in two years was attributed to lower rainfall received in the 2015/2016 and 2017/2018 agricultural seasons and associated crop competition for soil moisture. Less water stress and crop competition on the other hand was experienced in the higher rainfall season of 2016/2017 emphasizing again the strong influence of rainfall season on crop yields. We can therefore conclude that doubled-up legumes systems and their individual components are affected more seriously in drier seasons as a result of the competition from the constituent crops.

Legume intensification with pigeonpea and groundnut is therefore not overcoming climate related challenges, despite the drought-tolerant nature of both groundnut and pigeonpea. This is in contrast to results from Rusinamhodzi, et al.^46^ who concluded that this was the case for central Mozambique.

The maize grain and biomass yield differences between the seasons are also reflective of the different rainfall amounts received during the study period. There was no effect of preceding legume combinations on maize yields in subsequent seasons. The lack of response could be explained by (a) the duration of the trial as an accumulation of soil fertility (especially residual N and SOC) is a slow process and would require additional years to become apparent; (b) the current management of pigeonpea biomass in the system is critical. Traditional management of pigeonpea leaves and stems means cutting the pigeonpea stalks at harvest (in July/August) and replanting pigeonpea in the following cropping season. If this practice is continued, the green leaf of pigeonpea, the plant part where most of the nitrogen is accumulated^47^ will not be used and most of the N in the leaves will volatilize during the long dry period between harvest and planting maize losing the benefit to the atmosphere. Ratooning of pigeonpea at maize planting time and using the green leaves at seeding of maize would be a better strategy^48^; (c) the retention of fibrous biomass from pigeonpea may have led to negative effects on N mobilization. Although pigeonpea residue has a low C:N ratio as compared to many cereals, the high lignin content negatively influences its decomposition. This is further exacerbated by the retention of residues of the succeeding maize crop. Retention of crop residues with a wide C:N ratio can lead to nitrogen lock-up, depending on the soil type and rainfall regime^49,50^ which negatively affects the maize crop in subsequent years. Nitrogen lock-up has been associated with an increase in biological activity (macro- and meso-fauna) and proliferation of these organisms which limits available N for use by the plants^49,51^. Possibly as a result of these reasons, the residual effect of growing two legumes was not significant on individual maize yield in post-planted crops and confirms the rejection of our third hypothesis.

Total system grain yield was much influenced by the seasons. In 2016/2017, overall total system grain yield under both CA and RF out-yielded the performance of these systems in the other seasons (Fig. 4a). The effects of the treatments were masked by the seasonal effects. The 2015/16 season also showed the least overall yield, explained by the low rainfall received in this El Niño season. The greatest benefits on overall total system yield were observed in 2016/17 season where both pigeonpea and groundnut were grown (Fig. 4b). The favourable conditions of 2016/2017 favoured the systems with both legumes in various populations, which yielded highest. In the same year, the sub-treatment involving groundnut as a sole crop was comparable. Groundnut are known to be more drought-tolerant^52^ and are therefore a very important component in these systems. In all other years, the doubled-up legumes systems out-yielded sole legume systems. These results confirm that doubled-up legumes systems can provide farmers with additional calories to feed their families as well as their soil and livestock.

Increased levels of biomass as a result of doubling-up legumes or pigeonpea alone may be beneficial for CA systems in the longer term as retaining enough amounts of crop residues has been a major challenge in implementing CA systems in the region. Groundnut kernel yields were always higher in the trials as compared to pigeonpea grain yields, which indicates how important the crop is in this combination from a grain yield perspective. However, a greater proportion of biomass was produced by the pigeonpea even in 2015/2016 despite it being an El Niño year, confirming its drought-tolerance. Soil fertility benefits over time in the pigeonpea intercrop management systems have been reported and could reduce soil fertility decline as has been shown by various scholars^53–55^. Pigeonpea grain provides nutritional benefits and a potential cash income from the export markets. Stalks can be used as firewood or left standing and used as mulch at the onset of the cropping season while the leaves can be browsed by goats or cattle providing supplementary feed which is scarce during the dry season^20,21,36,56^.

The results of our study show that with ideal moisture levels and adequate pest management, doubled-up legumes systems may lead to yield advantage. The combined results of both legumes show that doubling-up legumes offers farmers a range of benefits and advantages over sole cropping^57^. In the study done by de Graaff and Kessler^29^ across districts of Malawi, pigeonpea-groundnut doubled-up legumes systems generated the greatest amount of biologically fixed N (82.8 kg N ha^−1^) as compared to sole cropping of pigeonpea (54.1 kg N ha^−1^) or groundnut (55.8 kg N ha^−1^).

For CA systems to become more attractive in the short term, doubled-up legumes systems may offer an additional entry point as they provide a diversity of marketable grain from legumes with different growth habits and sequence. In fact, groundnut is widely known and consumed by farmers in southern Africa, whereas pigeonpea has a good export market in India but is, to-date, less consumed by farmers. There is need to develop a local market and socialize the crop with farmers, so it is locally consumed.

Due to lack of funding and functional laboratory facilities on-site, the specific effects of doubled-up legumes systems under CA on soil fertility and biological nitrogen fixation, as well as economic benefits of the different systems could not be explored further. These remain future areas of research in the quest to find sustainable systems for smallholder farmers in Southern Africa.

## Conclusion

The research on doubled-up legumes systems under the conditions of eastern Zambia showed that there is a benefit of growing doubled-up legumes systems as compared to single crop rotations. We conclude from this research that: (a) the level of trial performance was strongly determined by the rainfall and cropping season and was not consistent; (b) there was no detectable residual benefit of doubled-up legumes systems on post-planted maize yield, which means that the contribution of preceding legumes to maize in the second cropping season is too small and this may be attributed to the short cycle annual rotation effect and/or the short experimental duration of 3 years which limited significant increase in soil fertility. Residual benefit of legumes on succeeding maize which is usually through soil fertility build-up depends on organic matter turnover and carbon sequestration which takes time; (c) Overall, total systems yield as well as total system grain and biomass yield can be improved by doubling-up the legumes and rotating with maize. This will provide not only additional grain yields but an improved nutritional supplementation for smallholders who are usually faced with low calorie intake; (d) supplementary biomass is produced by doubling-up legumes hence improving the levels of biomass in the system for surface crop residue retention as well as feed for livestock, which are an important component of smallholder farming systems; (e) growing doubled-up legumes systems under CA would potentially lead to reductions in labour for planting as CA systems can be flat planted without manual labour for ridging whereas the conventionally tilled system requires extensive labour on tilling and ridging the soil. This can potentially brighten prospects for CA. Further research should focus on the specific effects of doubled-up legumes systems under CA on soil fertility and biological nitrogen fixation, as well as on economic benefits.

## Materials and methods

### Study site description

The study was carried out in five on-farm communities of eastern Zambia (Table S1). Eastern Zambia lies between latitude − 10° to 15° S and longitude 30°–33° E^58^ and all farming systems practiced in this area are maize-mixed farming systems^59^. The sites lie in the Zambian agro-ecological zone IIa, which receives annual rainfall between 800 and 1000 mm^60^. The rainfall season is unimodal, usually starting in November and ending in April. The soils are relatively fertile *Acrisols* and *Luvisols*^61^.

### Experimental design

In establishing the doubled-up legumes systems trial, only combinations of legumes were planted to plots in the first season (the legumes phase). In the second season, the legumes phase plots were superimposed with a uniform maize crop (the maize phase) and a new legumes phase was equally added. In the third season, the plots hosting the legumes combinations were once again super-imposed with a uniform maize crop while a second legume phase was initiated on plots hosting the uniform maize crop in the previous season. In summary, plots planted to legumes combinations were planted to a uniform maize crop in the subsequent season and alternated thereafter. However, the trial could not proceed beyond the third season due to funding constraints.

The trial was set up in a split plot design with five replications at each site. The main treatments were based on crop management systems:

1. Conservation Agriculture (CA)—no-tillage with residue retention; and
2. Conventional tillage-based ridge and furrow tillage (RF)—with residue removal

The main plots were split into sub-plots with four sub-treatments based on different legumes with different populations:

1. Groundnut planted as sole crop (soleGn)
2. Pigeonpea planted as sole crop (solePp)
3. Groundnut planted in full population plus half population of pigeonpea (fullGN/halfPP)
4. Groundnut planted in full population plus full population of pigeonpea (fullGN/fullPP)

The plot size was 6 m × 6 m and a net plot of four rows by 5 m in length was harvested. Land preparation for the RF treatment was in manually dug ridges, prepared in October before the onset of the cropping season. The ridges were spaced at 75 cm and had a height of approximately 25–30 cm. Sowing under CA was done in riplines of approximately 10 cm depth created by an ox-drawn *Magoye* ripper without further soil disturbance.

### Crop management

Crops were sown after the first effective rains at each site which was in mid-December of each season. In the first season, the groundnut variety MGV 4 and pigeonpea variety Chitedze 1 were sown. In RF, groundnut was sown in a double row spaced at 10 cm apart on top of ridges spaced at 75 cm and with an in-row spacing of 20 cm (target population of 133,333 plants ha^−1^). In CA systems, groundnuts were sown in rows spaced at 37.5 cm on the flat with an in-row spacing of 20 cm (133,333 plants ha^−1^). Although farmers are currently only sowing one row of groundnut on top of the ridge in the RF system, we used double rows to avoid higher plant populations in the CA system as the plant population would double and possibly bias the results in favour of CA. Pigeonpea in full population (sub-treatments 2 and 4) was sown at a spacing of 75 cm × 60 cm with 2 plants per station (44,444 plants ha^−1^) whereas in its half population (sub-treatment 3), it was sown with only one plant per station (22,222 plants ha^−1^). Pigeonpea was sown either on top of the ridge between the groundnut rows (RF) or planted in every second groundnut row when planted on the flat (CA). Maize sowing in the season following the legumes phase was done at a plant spacing of 75 cm × 25 cm (53,000 plants ha^−1^) for all treatments. The commercial, medium maturing maize variety KKS501 was sown in both cropping seasons.

Fertilization was uniform across all treatments at a rate of 10 kg ha^−1^ N:20 kg ha^−1^ P_2_O_5_:10 kg ha^−1^ K_2_O applied as basal dressing at sowing to both legumes and maize in each season. This is half the Zambian Government recommended rate. Maize further received a top dressing of 46 kg ha^-1^ N in the form of urea which is again half of the recommended rate. Legumes also received gypsum at a rate of 500 kg ha^−1^ (147 kg ha^−1^ Ca and 118 kg ha^−1^ S) to enhance pod formation. Weed control at all sites was achieved with an initial spray of glyphosate [*N*-(phosphono-methyl) glycine] at a rate of 3 l ha^−1^ followed by hoe weeding whenever weeds reached 10 cm in height or circumference for weeds with a stoloniferous growth habit. An initial application of 2.5–3 t ha^−1^ (dry-weight) of maize stalk residues was done to the first-season legume plots and at the beginning of each season thereafter. Pest control was minor in the first season but pests such as the blister beetle (*Mylabris oculata* MYLBPU) and cotton bollworm (*Helicoverpa amigra* Hb.) heavily attacked the pigeonpea which led to a significant yield penalty on the crop in the first season (See Figure S1). We therefore resorted to spraying the pigeonpea with DDVP (Dichlorvos; active ingredient 2,2-dichlorovinyl dimethyl phosphate) at recommended rates to control these insects from the second season onwards. Relevant institutional, national, and international laws guiding studies on plants were adhered to in the conduct of this study.

### Data collection

Rainfall collected in rain gauges installed at each site was recorded daily by farmers who hosted the trial after every rainfall event (usually before 9:00 a.m.). Yields were determined from the net plots by weighing all the plants including the pods or cobs, removing the pods or cobs and weighing them separately. Subsample of pods (approximately 500 g) or 10 cobs and biomass (approximately 500 g) were taken to determine the dry weight. After shelling the subsamples and weighing the grains, a moisture reading was taken (using a mini GAC moisture tester from Dickey – John, USA) to determine the yield at 12.5% and 9% moisture content for maize and legumes, respectively. Biomass weight was determined at a dry-weight basis. Both yields were then reported in t ha^-1^.

### Calculations and statistical analyses

#### Legume and maize productivity and total grain, total biomass and overall total system yield

To assess the effects of crop management systems and different legumes and their different population combinations on yield of maize, yield of legumes, and total system yield; mixed models were used for the split plot design. In these models, crop management systems had the main effects while the different legumes and their different populations had the sub-effects, and these were included as fixed effects together with seasons (years). Sites and plots within blocks within sites were included as random effects to account for grouping factors and repeated measures across the seasons. The significance of the fixed effects was tested using the Wald chi-square test in Asreml-R package^62^ as well as using the 'lme4’ package^63^ and mean contrasts were done using the multiple comparison procedure with multiplicity adjustment using the 'emmeans' package^64^ in R statistical environment^65^. Overall total system yield was calculated as a sum of both grain yield and biomass yield of all crops involved in each crop management system converted to energy in GJ ha^−1^. For the conversion of grain yield to energy, grain energy values were obtained from the GENuS database where energy values of the unprocessed grain are reported. Maize, pigeonpea and groundnut are indicated to contain 353 kcal 100 g^−1^, 301 kcal 100 g^−1^ and 578 kcal 100 g^−1^, respectively. For the energy values of above ground biomass, we referred to various sources such as the Feedipedia (https://www.feedipedia.org/) where energy values of biomass were reported for livestock feeding. Maize, pigeonpea and groundnut biomass were reported to contain 125 kcal 100 g^−1^, 186 kcal 100 g^−1^ and 155 kcal 100 g^−1^ of energy in their biomass, respectively. Energy values of each system were calculated using either Eq. (1) or (2) as follows:

1 $${TS}_{ij} = \frac{\left({Yc}_{i}\right)\left({Ec}_{i}\right)\left(10\right) + \left({Yc}_{j}\right)\left({Ec}_{j}\right)(10) }{{GJ}_{conv}}$$

2 $${TS2}_{ij} = \frac{\frac{1}{2}\left[\left({Yc}_{i}\right)\left({Ec}_{i}\right)\left(10\right)\right] + \frac{1}{2}[\left({Yc}_{j}\right)\left({Ec}_{j}\right)\left(10\right)] }{{GJ}_{conv}}$$

where TS_*ij*_* and TS2*_*ij*_ are the total system yield of crops in the system, with respect to their energy value (GJ ha^−1^) for systems without rotation and with rotation, respectively, that involved crops *i* and *j*. Yc_*i*_ and Yc_*j*_ are yields of crops in each phase of the system (kg ha^−1^; Ec_*i*_ and Ec_*j*_ represent energy contents of crops i and j involved in the intercrop (kcal 100 g^−1^) and GJ_*conv*_ is a conversion factor that converts kcal to GJ, where 1 GJ is 238,845.9 kcal. Equation (1) was used for calculation of total system yield in season 2015/16 since only the legume phase of the rotation was planted and Eq. (2) was used for seasons 2016/17 and 2017/18 since both the maize and legume phases of the rotation were present and yield was based on half of each system in each phase.

### Land equivalent ratio

Data was further presented in terms of land equivalent ratio (LER) to better understand the ratio of the area under legume sole cropping to the area under companion legumes systems that would be needed to produce the same total system yield^66^. The LER was used to assess the total system yield advantage of the different legume intercropping configurations based on crop management system and season as compared to the monocropping system. Since two intercropping systems were involved based on different populations of the legumes, LER was assessed separately for these i.e., (a) ratio of sole cropping to intercropping with full populations of groundnut and pigeon pea and, (b) ratio of sole cropping to intercropping with full groundnut population plus half population pigeonpea. Land equivalent ratio was calculated using Eq. (3):

3 $$\mathrm{LER total}=LGn+LPp= \frac{{Y}_{dGn}}{{Y}_{sGn}}+\frac{{Y}_{dPp}}{{Y}_{sPp}}$$

where LGn and LPp are partial LERs for groundnut and pigeonpea, respectively; Y_*dGn*_ and Y_*dPp*_ are the energy values of groundnut and pigeonpea in the companion system, respectively (GJ ha^−1^); Y_*sGn*_ and Y_*sPp*_ are energy values of sole groundnut and pigeonpea, respectively (GJ ha^−1^). If the LER is greater than 1, it means that there is an advantage of the companion legumes system over sole planting and if it is below then there is a yield penalty in using the companion legumes system as compared to sole cropping. Effects of crop management systems and seasons on LER were analysed using the same models as used for yield in the previous section.

## Supplementary Information

Supplementary Information.

## Data Availability

Data used in the study is available upon request.

## Code availability

The R code used in the study is available from the corresponding author upon reasonable request.
