# Supplementary material for: Intensifying cropping systems through doubled-up legumes in Eastern Zambia
Source: Sci Rep. 2021 Apr 14;11:8101. doi: 10.1038/s41598-021-87594-0 (PMC8047045; doi:10.1038/s41598-021-87594-0)
Supplement: Supplementary file 1 — Supplementary Information. [file 41598_2021_87594_MOESM1_ESM.pdf]

Supplementary material

Intensifying cropping systems through doubled-up legumes in Eastern Zambia

Mulundu Mwila[1]*, Blessing Mhlanga[2] and Christian Thierfelder[3]

[1]* Corresponding author: Zambia Agriculture Research Institute, Msekera Research Station, Chipata, Zambia, +260977509855, mulundu.mwila@agriculture.gov.zm ORCID iD: 0000-0001-6234-5408

[2]Institute of Life Sciences, Scuola Superiore Sant'Anna, Piazza Martiri della Libertà 33, 56127 Pisa, Italy, +393791589843, b.mhlanga@santannapisa.it ORCID iD: 0000-0003-4587-795X

[3]International Maize & Wheat Improvement Centre (CIMMYT), Harare, Zimbabwe, +263772815230, c.thierfelder@cgiar.org ORCID iD: 0000-0002-6306-7670

1. Tables

Table S1. Brief description of the five study communities; Hoya, Kapara, Mtaya, Chanje and Kawalala, Eastern Zambia

District	Community	Longitude	Latitude	Altitude	Soil type	Farming system
Sinda	Kawalala	-14.0953	31.48860	934	*Acrisols*	Maize mixed
Chipata	Chanje	-13.2330	32.47892	882	*Luvisols*	Maize mixed
Chipata	Kapara	-13.3013	32.29310	711	*Luvisols*	Maize mixed
Chipata	Mtaya	-13.3438	32.31201	788	*Luvisols*	Maize mixed
Lundazi	Hoya	-12.0715	33.07986	1096	*Acrisols*	Maize mixed

2. Figures

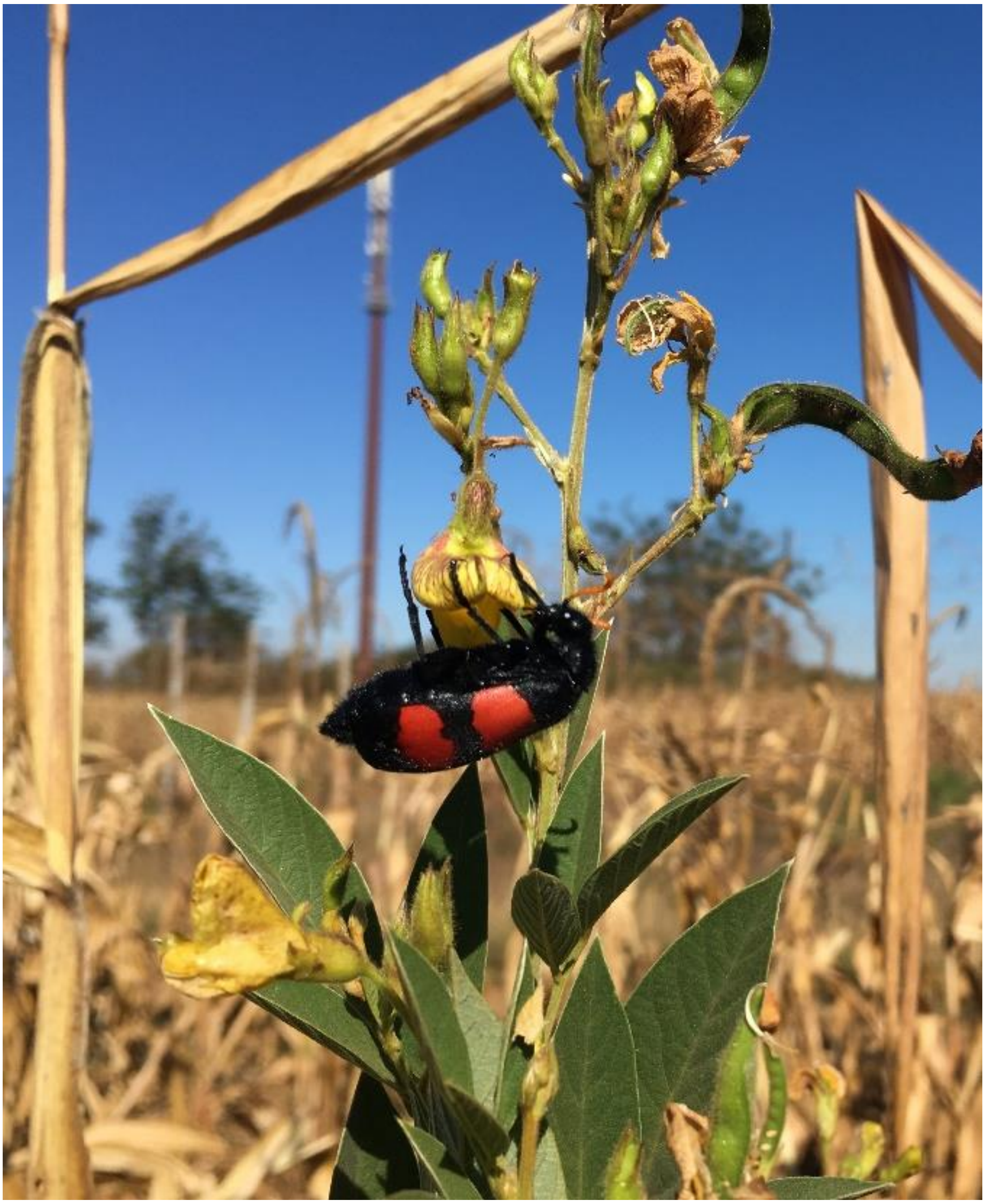

Figure S1. A blister beetle attacking pigeonpea. Photo credit: Thierfelder, CIMMYT

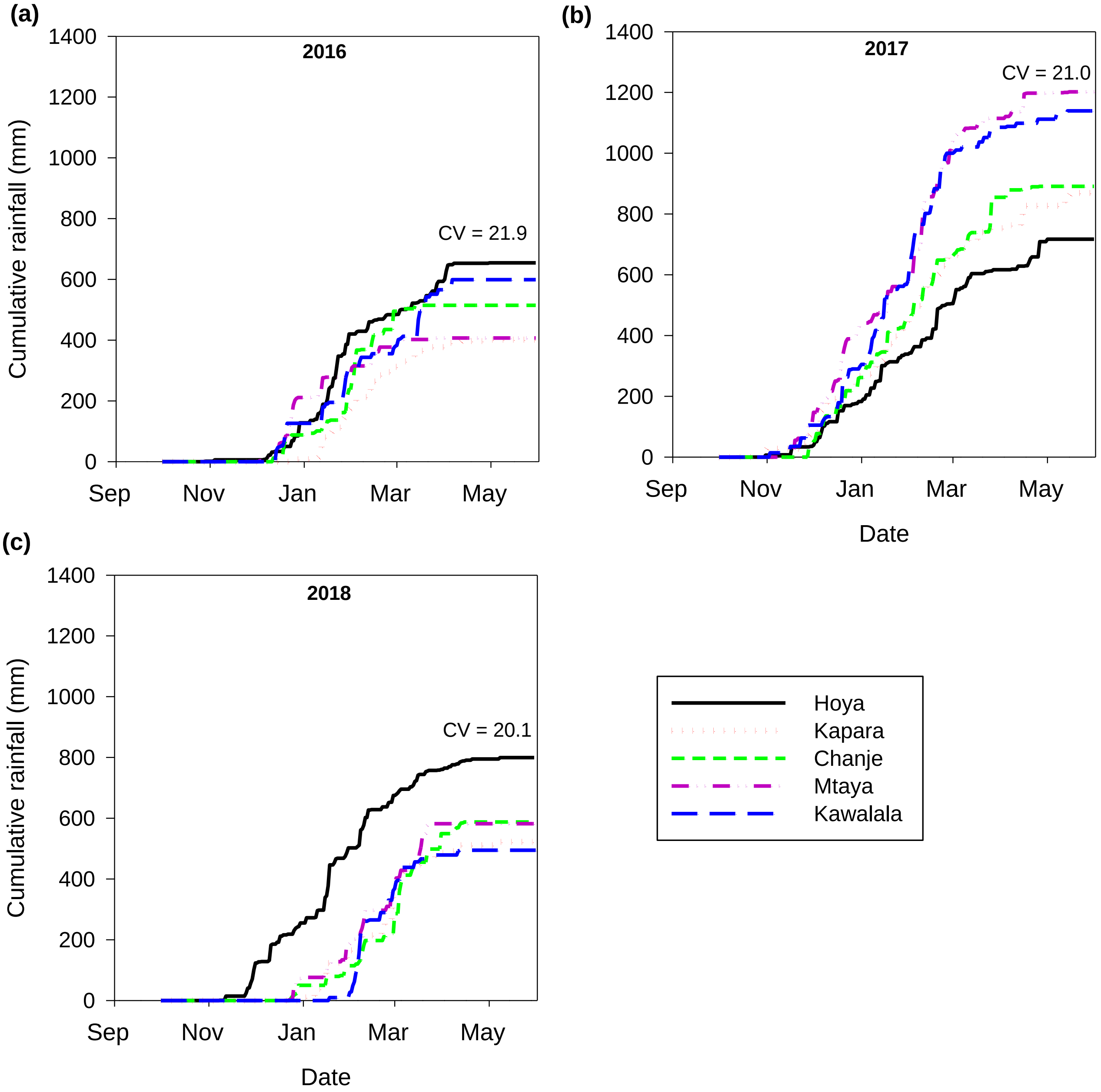

Figure S2. Cumulative rainfall in the five target communities where doubled-up legume systems were carried out in the (a) 2016/2017, (b) 2017/2018 and (c) 202018/2019 agricultural seasons in Eastern Zambia. The within-season coefficients of variation (CVs) between the communities are given for each season.
